# Bilateral synovial chondromatosis in the knee joint with both intra and extra-articular diseases

**DOI:** 10.11604/pamj.2014.19.57.4054

**Published:** 2014-09-23

**Authors:** Rida-Allah Bassir, Farid Ismael, Ahmed Elbardouni, Mustapha Mahfoud, Mohamed Saleh Berrada, Moradh Elyaacoubi

**Affiliations:** 1 Department of Orthopaedic Surgery and Traumatology, Mohamed V, Souissi University, Rabat, Morocco

**Keywords:** Synovial chondromatosis, knee, bilateral, literature

## Abstract

Synovial chondromatosis is a rare disease of unknown etiology. It usually occurs unilaterally in the large joints like the knee, but may occur in the shoulder, elbow, hip, ankle and temporomandibular joints. The disease is usually intracapsular, but can also be extracapsular on rare occasions. The diagnosis of synovial chondromatosis is given after an anamnesis, physical examination and radiographic examination. However, the diagnosis is obtained after histological examination of the synovial tissue. We report an unusual presentation of bilateral synovial chondromatosis in the knee joint, with both intra and extracapsular localization. never described in the literature. Although synovial chondromatosis is described as a benign disease, it can be very destructive and debilitating. These lesions can mimic a malignant tumor and present a diagnostic problem.

## Introduction

A synovial chondromatosis is a rare benign neoplasm that is caused by metaplasia of the synovium into chondrocytes [1]. The aetiology of the disease is uncertain. Milligram classified the disease into three phases: early(active intrasynovial disease but no loose bodies), transitional disease (active disease and loose bodies), and late (multiple loose bodies but no intrasynovial disease) [2]. The disease is commonly mono-articular and mostly affects the knee [3]. It occurs twice as frequently in men than women and usually presents with increasing joint pain and swelling during the third to fifth decade of a patient's life. A patient with synovial chondromatosis experiences a decreased range of motion, palpable swelling,effusion, and crepitus [4]. The disease is usually intracapsular, but can also be extracapsular on rare occasions [5]. In this case report, we describe a patient with both intra- and extra-articular diseases affecting both knees. To the best of our knowledge, this is the first case with such an extensive presentation of intra and extra-articular disease affecting bilateral the knee joint.

## Patient and observation

**Figure 1:**
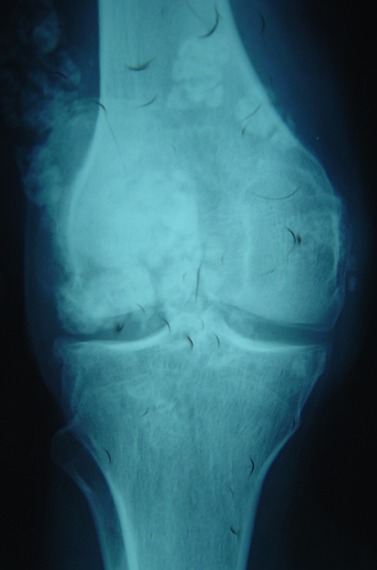
Frontal plain radiograph showing multiple soft tissue calcifications within and outside the joint capsule of the right knee

**Figure 2:**
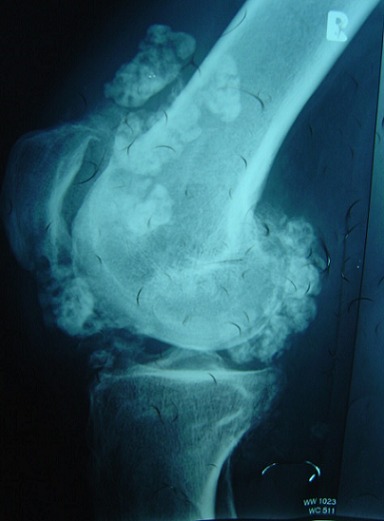
Lateral plain radiograph showing multiple soft tissue calcifications within and outside the joint capsule of the right knee

**Figure 3:**
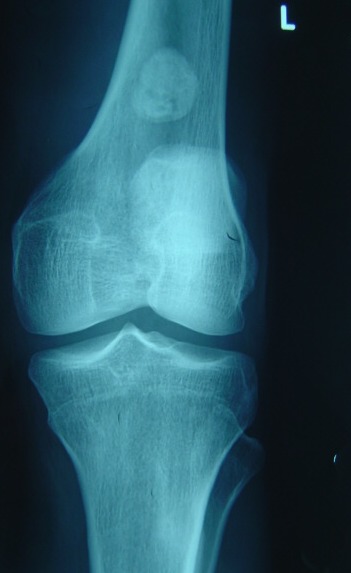
Frontal plain radiograph showing a solitary calcification on the left knee

**Figure 4:**
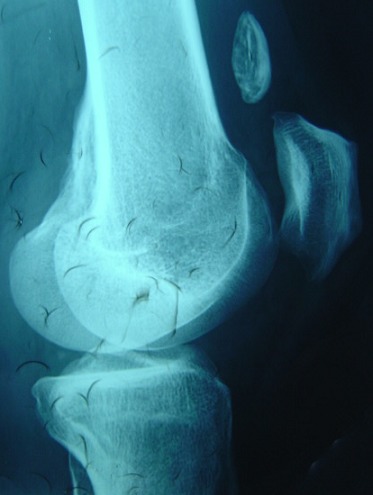
Lateral plain radiograph showing a solitary calcification on the left knee

A 38-year-old man presented with a seven month history of progressively worsening bilateral knees pain with associated swelling. The pain was present when the patient was at rest, and worsened when the legs was bearing weight, thus restricting his walking to short distances. His knees had become increasingly swollen. He was otherwise fit and well. His medical history was unremarkable and he was only taking a paracetamol, codeine and anti-inflammatory drugs for the pain. Upon examination, the patient was seen to have visibly swollen popliteal fossa and marked quadriceps wasting of his right lower limb. On palpation, the masses was hard, mobile, well defined, and measured 0.5 - 04 cm. The swelling was non-tender and there were no associated skin changes. He could fully extend his knee, but flexion was restricted to only 110 degrees. There was a McMurray test proved equivocal and no ligamentous instability. An examination of the patient's hip revealed no abnormality. A plain radiograph of the patient's knees revealed multiple calcific densities within the soft tissues surrounding it on the right one (Figure 1, Figure 2). Although some of these appeared to lie within the capsule, the majority appeared to be outside of it, and a solitary image on the left knee (Figure 3, Figure 4). These appearances were thought to be consistent with idiopathic tumoral calcinosis. However, to further scrutinize these calcifications, a magnetic resonance imaging (MRI) scan was recommended. It showed an extensive thickening of the patient's synovium, multiple intraarticular calcific and ossific loose bodies, and large calcified bursal extensions. These findings were thought to be consistent with very extensive bilatéral synovial chondromatosis.

**Figure 5:**
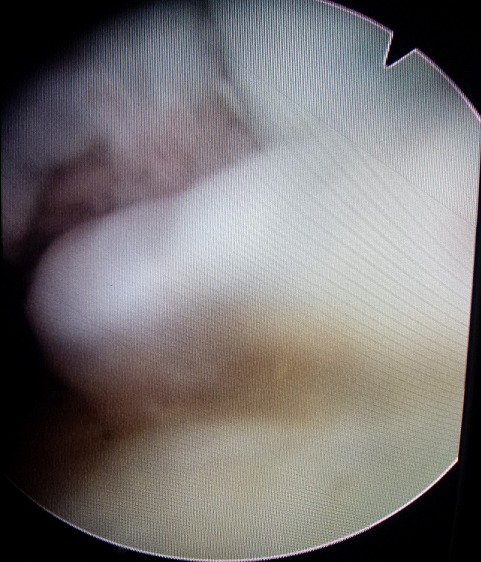
An arthroscopic photograph showing nodules of chondromatosis fixed to the synovium

**Figure 6:**
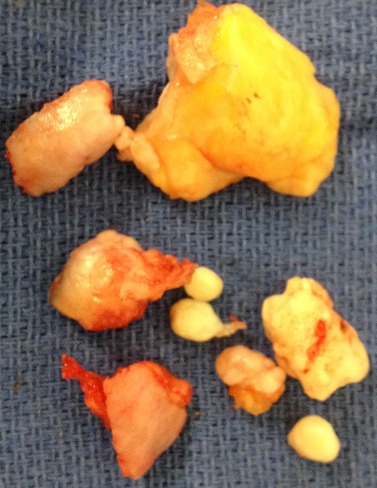
Excision of synovium and nodules

The patient's blood tests were normal: C-reactive protein 5 mg/l and the phosphate calcium balance without errors. A two-stage procedure was planned following the findings of the MRI scan. The first stage was arthroscopy (Figure 5), which was able to note Grade IV osteoarthritis alongside florid synovial chondromatosis in the lateral compartment of the right knee. There were multiple loose bodies within this compartment and nodules were fixed to the synovium. On the left one,we found an isolated synovial metaplasia in the subvastus quadriceps-sparing. A synovectomy with debridement and excision of these bodies was thus performed (Figure 6). The second stage involved an open exploration of the patient's popliteal fossa. Multiple calcified masses were found, all enclosed in bursal sacs. They were lateral to the semimembranosus at the level of the oblique popliteal ligament. All the masses were excised. A histological review confirmed our diagnosis of synovial chondromatosis. The sections showed nests of chondrocytes with focal ossification and focally attenuated synovium overlying the nodules. After the operation, the patient underwent functional rehabilitation sessions focusing on quadriceps strengthening, with a daily exercise regime to supplement this. He recovered well and ten weeks after the operation,has regained his right knee's full range of movement with flexion increased to 130 degrees, which is equal to that of his left knee.

## Discussion

Synovial chondromatosis is a rare benign condition characterized by the presence of cartilaginous nodules in the synovium of joints, tendon sheaths, and bursae which often occur without trauma or inflammation [1]. With disease progression, the loose bodies may ossify and can be identified radiographically [6]. There are a variety of names for this lesion. The most commonly accepted include synovial chondromatosis, synoviochondrometaplasia, synovial chondrosis, synovial osteochondromatosis, and articular chondrosis [6]. The condition is generally thought to be monoarticular and over 50% of reported cases occur in the knee [7]. Extra-articular synovial chondromatosis is rare, but the combination of intra- and extra-articular diseases described here is an extremely rare condition. Given the initial X-ray image of large extra-articular calcification,we felt that the patient was more likely to have idiopathic tumoral calcinosis. However, tumoral calcinosis usually only affects people from Africa and the Caribbean in their second decade of life [8]. Extra-articular diseases can be classified as tenosynovial chondromatosis or bursal chondromatosis depending on the origin [9].

It is generally agreed that the exact aetiology of synovial chondromatosis is unknown and controversy exists surrounding proposed hypotheses. Milgram, in 1977, categorized the disease process into 3 distinct phases [10]. In phase I, metaplasia of the synovial intima occurs. Active synovitis and nodule formation is present, but no calcifications can be identified. In phase II, nodular synovitis and loose bodies are present in the joint. The loose bodies are primarily still cartilaginous. In phase III, the loose bodies remain but the synovitis has resolved. The loose bodies also have a tendency to unite and calcify [10]. Because there is no evidence of histologic metaplasia in stage three, diagnosis may be more difficult. To the best of our knowledge, this pattern of disease in the knee has never been reported in the literature. This obviously raises concerns regarding a possible transformation to synovial chondrosarcoma. However, histological investigation revealed no significant nuclear atypia, thus ruling out malignancy. The literature reports only 33 cases of malignant transformation in the setting of histologically confirmed synovial chondromatosis [7]. A key feature of all these cases is the recurrence of benign disease prior to a diagnosis of malignant disease. The diagnosis of synovial chondromatosis is often made following a thorough history, physical examination, and radiographic examination. Patients may report pain and swelling within a joint. This is routinely exacerbated with physical activity. Commonly, the patient may also report aching, reduced range of motion, palpable nodules, locking, or clicking of the joint [6]. These lesions may become symptomatic following mechanical compression or irritation of soft tissues, nerves, or malignant transformation. In rare cases, reactive bursas can form over osteochondromas. These may be another source of pain, but can also mimic chondrosarcoma.

Conversely, individuals may have no signs or symptoms and it is an incidental finding secondary to another complaint. According to Milgram, this is related to the stage of the lesion [10]. According to Milgram′s classification, plain film radiographs are only helpful in the third phase of the disease, once calcification has occurred [10]. Advanced imaging, such as CT and MRI scans are useful in identifying and localizing the lesions as well as helping to distinguish between other differential diagnoses. Blood tests and arthritis profiles can also help rule out specific differential diagnoses. As radiotherapy and chemotherapy have no effect on synovial chondromatosis, surgical excision is the preferred treatment [4]. In cases that involve localized intra-articular disease, complete excision of the abnormal synovium seems to provide a cure. Generalized intra-articular disease with pain and swelling requires total synovectomy and a removal of the loose bodies. Extra-articular disease treatment aims for complete excision [8].

## Conclusion

This case is reported because of its rarity. No other cases of bilateral knee synovial chondromatosis with intra and extra capsular localisation, have been reported. Highlights the importance of careful clinical assessment, appropriate use of investigation, and careful pre-operative planning.
